# Development of interactive empowerment services in support of personalised medicine

**DOI:** 10.3332/ecancer.2014.400

**Published:** 2014-02-11

**Authors:** Haridimos Kondylakis, Eleni Kazantzaki, Lefteris Koumakis, Irini Genitsaridi, Kostas Marias, Alessandra Gorini, Ketti Mazzocco, Gabriella Pravettoni, Danny Burke, Gordon McVie, Manolis Tsiknakis

**Affiliations:** 1 Computational Medicine Laboratory, FORTH-ICS, Heraklio, Crete GR-71110, Greece; 2 Department of Decision Sciences, Bocconi University, Milan 20136, Italy and Cancer Intelligence Ltd, 154 Cheltenham Road, Bristol, BS6 5RL, UK; 3 Department of Applied Informatics and Multimedia, Technological Educational Institute, Heraklion, Crete GR-71004, Greece

**Keywords:** patient empowerment, psycho-cognitive models, personal health record

## Abstract

In an epoch where shared decision making is gaining importance, a patient’s commitment to and knowledge about his/her health condition is becoming more and more relevant. Health literacy is one of the most important factors in enhancing the involvement of patients in their care. Nevertheless, other factors can impair patient processing and understanding of health information: psychological aspects and cognitive style may affect the way patients approach, select, and retain information. This paper describes the development and validation of a short and easy to fill-out questionnaire that measures and collects psycho-cognitive information about patients, named ALGA-C. ALGA-C is a multilingual, multidevice instrument, and its validation was carried out in healthy people and breast cancer patients. In addition to the aforementioned questionnaire, a patient profiling mechanism has also been developed. The ALGA-C Profiler enables physicians to rapidly inspect each patient’s individual cognitive profile and see at a glance the areas of concern. With this tool, doctors can modulate the language, vocabulary, and content of subsequent discussions with the patient, thus enabling easier understanding by the patient. This, in turn, helps the patient formulate questions and participate on an equal footing in the decision-making processes. Finally, a preview is given on the techniques under consideration for exploiting the constructed patient profile by a personal health record (PHR). Predefined rules will use a patient’s profile to personalise the contents of the information presented and to customise ways in which users complete their tasks in a PHR system. This optimises information delivery to patients and makes it easier for the patient to decide what is of interest to him/her at the moment.

## 1. Introduction

It is self-evident that it is important for a doctor to understand who the patient is as he or she is entering the doctor’s office, as the doctor needs to provide that patient with information first hand that is not only accurate but also personalised and tailored to the patient’s educational level. Up to 50% of patients are sceptical of the communication aspects of their hospital care, even when clinicians think they have made special efforts to communicate well [1]. Among other issues, a proportion of patients consider that their opinion is ignored and they are not able to make decisions about their health care. In general, patients want more information than they are given [2]. Since patients vary in their existing knowledge and the amount and type of information they want as well as their expectations of the consultation, it is important to discuss these with them. It is also essential to check the comprehension and recall of relevant information with patients at regular intervals. A common communication problem is the use of jargon and technical terms.

On the other hand, some groups of patients are more difficult to handle for the clinicians than others, so they have to develop appropriate strategies to cope with them. These groups are, for example, ethnic minorities, young patients, or elderly people. Communication also becomes harder with patients who have mental health or drug problems. Good communication could be very beneficial for patients, and it would enhance clinical outcomes in a cognitive, affective, and behavioural way.

To this aim, the ALGA-C questionnaire has been developed. It aims to provide a psycho-cognitive picture of the patient whom the physician is about to meet, so that (s)he will use the feedback from ALGA to optimise his communication style and catalyse the interaction. More specifically, while waiting for the medical visit, the patient will fill in the ALGA-C questionnaire on an electronic device connected with the patient’s personal health record (PHR). The outcome of the questionnaire is immediately stored and recoded in a patient’s profile that is used by the physician to adjust content, level, and modalities of verbal information to the patient’s needs. On the clinician’s side, the ground-breaking element provided here is the possibility to have access to the data that is not just medical, but rather centred on the values and needs of the specific patient, through the profile originated from an ALGA-C. The combined clinical and psycho-cognitive information becomes patient’s and doctor’s common knowledge, around which they can build together long term efficient decision-making plans.

However, ALGA-C is not a questionnaire thought to remain isolated for the health system, but part of a larger platform, the so-called interactive empowerment service (IEmS) platform. This platform is developed within the European Union p-medicine project [1], a research project that aspires to create an infrastructure that will facilitate the translation from current medical practice to personalised medicine. In the aforementioned platform, clinical information is patient-tagged and combined with psychological, social, and cognitive characteristics from patients. This information is then available throughout the entire platform to be used.

Another key component, which will use the aforementioned profiling information, is a PHR system with double importance. First, it is a container of all the information related to the patient, such as information on diagnosis and bio-bank data, as well as information on possible related clinical trials. Second, the patient has the opportunity to have access to such information and can be offered the opportunity to adopt responsibility for his medical record, including decisions on who should see which data. In the PHR, the patient can follow the journey of his/her data, and based on it, begin the decision process. However, to have ‘high-quality’ health information presented to a patient, it is not sufficient that its content is accurate. It is also necessary that accurate content is comprehensible to the person who has to use that information. That’s why we focus on intelligent optimisation of PHR based on patient profiling information. More specifically, techniques of adaptive presentation and adaptive navigation are explored in parallel with smart recommendation and intelligent alerts.

The rest of this paper is structured as follows: Section 2 presents the ALGA-C questionnaire and the profiler applications. Then, Section 3 presents how the questionnaire was validated with healthy people and breast-cancer patients. Section 4 presents out current explorations on the optimisation of PHR system according to the patient profile constructed using the aforementioned questionnaire. Finally, Section 5 concludes this paper and discusses future directions.

## 2. The ALGA-C questionnaire & the ALGA-C profiler

The ALGA-C questionnaire is a web-based tool [2] developed to support patient profiling and it is presented in details in other publications [3, 4]. The three macro-areas under consideration are (a) cognitive, (b) physical-related, and (c) psychological aspects, all investigated by different sub-dimensions.

**Figure 1.**
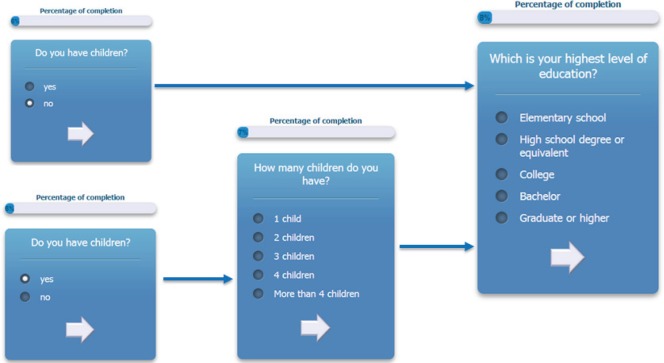
An example of the answer-based question flow.

**Figure 2.**
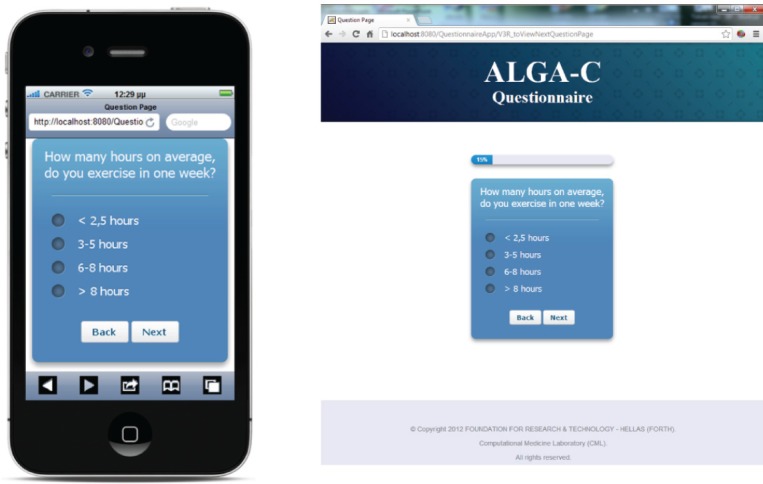
Screenshots from the questionnaire on an iPhone and on a desktop PC.

The questionnaire is multilingual; it is currently translated in English, Italian, Spanish, French, German, and Japanese, and the flow of the questions depends on user answers. An example of the answer-based question flow of the ALGA-C questionnaire is shown in Figure 1. In addition, the questionnaire is developed to be compatible with a variety of mobile devices and browsers. It is optimised for the following mobile devices: iPhone, iPad, Nexus 7, Nexus 4, and for the following web browsers: Chrome, Safari, Firefox, Opera, and Internet Explorer. Example screenshots of the application are shown on Figure 2.

**Figure 3.**
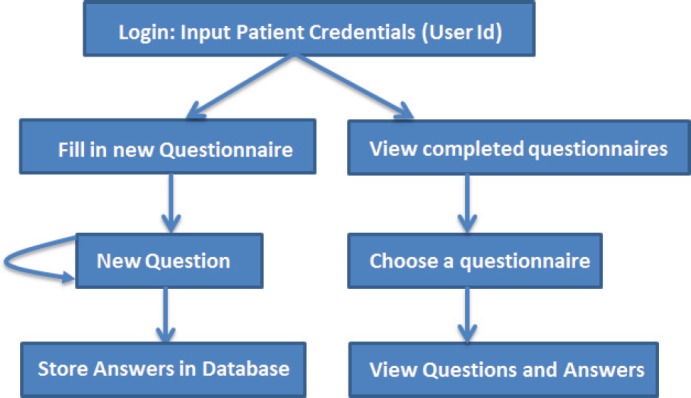
The basic workflow of the ALGA-C questionnaire.

The basic workflow of a patient filling in the questionnaire is shown in Figure 3. A patient logs into the system, and then he is able to view his past completed questionnaires, or he can fill in a new questionnaire. Each time he completes a questionnaire, the results are stored in a local database.

The back-end of the application is based on the Spring MVC 3 framework, Apache Tomcat server, and MySQL database. In addition, the interaction of the application with its database is facilitated by the Java Persistence API (JPA) EclipseLink. The front-end of the application is based on Java Server Pages, HTML5, and JQuery technologies. In addition, the application guarantees responsiveness by relying on the responsive CSS Framework and Twitter Bootstrap.

**Figure 4.**
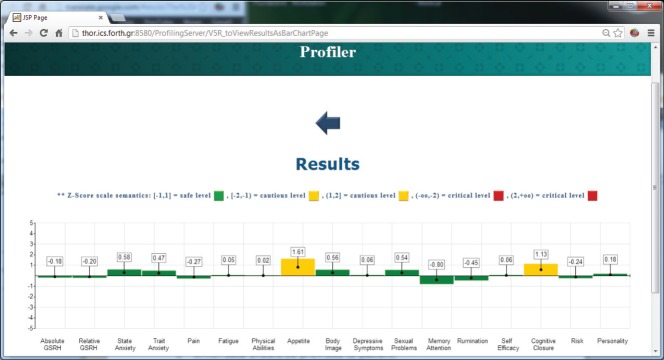
The results of the psychological analysis as a bar chart.

**Figure 5.**
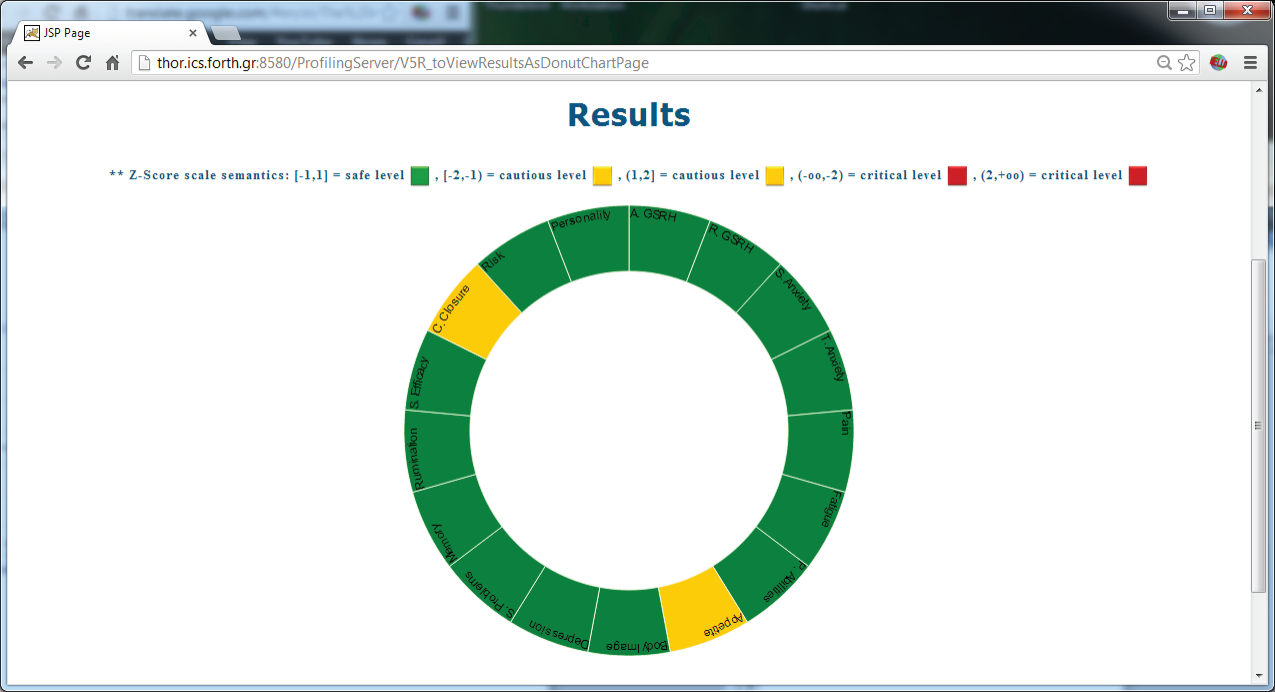
The results of the psychological analysis as a donut chart.

The ALGA-C Profiler on the other hand, analyses the patients’ answers from the ALGA-C questionnaire to extract their psychological profiles. The patients’ profiles are then available to the clinicians in multiple alternative visualisations through the profiler, shown in Figures 4 and 5. This knowledge facilitates the patient–clinician relationship in their communications and shared decision making. The profiling analysis is based on the answers that a patient has given in the ALGA-C questionnaire. The analysis of the results is conducted in two phases. The first phase contains the calculation of simple average scores. These scores are calculated based on the fact that each question’s answers have different weights defined by the p-medicine psychologists. The second phase of the analysis contains the calculation of Z-scores, which are more complex, as they consider standard deviations of a healthy group of patients that have already completed the questionnaire. The results of the analysis are provided to the clinicians in multiple alternative visualisations for facilitating their interaction in the decision-making process.

## 3. ALGA-C validation

The validation activities for the ALGA questionnaire started with an item pool generation, using questions selected from a number of validated psychological questionnaires. The initial draft of the ALGA questionnaire contained 46 items related to four themes of physical and psychological health status plus an initial set of 20 demographic questions and a final set of six questions regarding the difficulties eventually encountered by the subjects in filling out the questionnaire.

The investigation of norm scores from the general population facilitates the specification of cut-off scores necessary for referral.

For this first aim, the questionnaire has been administered to 778 healthy control subjects (who have never received a diagnosis of cancer).

Furthermore, the comparison of scores from healthy control subjects in the general population and cancer patients provides information about the discriminant power of the instrument concerning the detection of differences in scores of clinical and non-clinical individuals.

For this second aim, 45 women operated for newly diagnosed primary breast cancer in Italy formed the first pilot group.

Healthy control subjects completed the questionnaire online, while patients answered the questions using an iPad while waiting for the first appointment with the medical oncologist after the operation.

The total sampling population (*n* = 823) was analysed using the factor analysis [5], a perspective on how to study the human mind that has become very influential in modern psychology. Specifically, the exploratory factor analysis (EFA) was analysed by the principle component extraction method with VARIMAX rotation [6] to examine the relationships among variables without determining a particular hypothetical model. The items whose loading value was 0.45 or over were kept. Internal consistency was assessed by calculating the Cronbach’s alpha coefficient [6].

Considering healthy control subjects, the age mode was 40–49 years (23.5%). Sixty-five per cent were female, and 24.7% had undergone a psychological treatment in their lives. Most of the subjects were married (49.7%) and had diplomas (99%).

For the patients, the age mode was 40–49 years (35.6%). All of them were female, and 17.8% has undergone a psychological treatment in their lives. Most of the subjects were married (75.6%) and had diplomas (73.3%).

**Figure 6.**
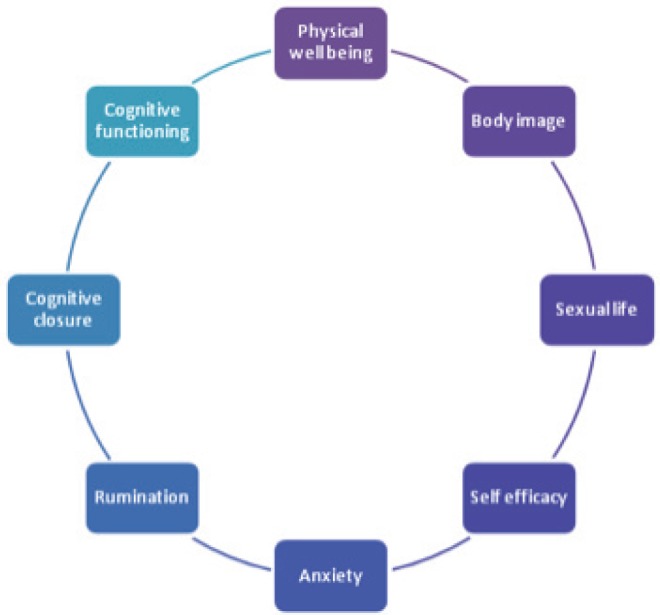
The eight factors composing the ALGA questionnaire which explain the personal profile.

**Figure 7.**
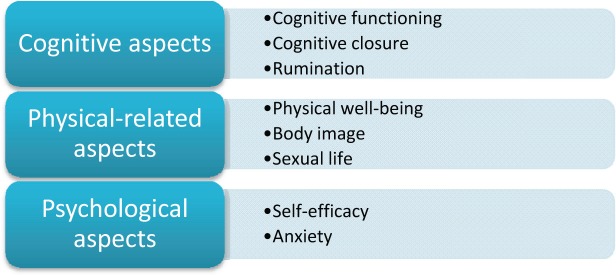
The three macro-areas ‘cognitive aspects’, ‘physical-related aspects’, and ‘psychological aspects’, in which the eight factors are categorised.

The factor analysis individuated the following components as relevant in explaining the personal profile: ‘physical well-being (F1)’, ‘body image (F2)’, ‘sexual life (F3)’, ‘self-efficacy (F4)’, ‘anxiety (F5)’, ‘rumination (F6)’, ‘cognitive closure (F7)’, and ‘cognitive functioning (F8)’ being assigned to the set of variables with high loadings on the first eight factors (Figures 6 and 7).

**Figure 8.**
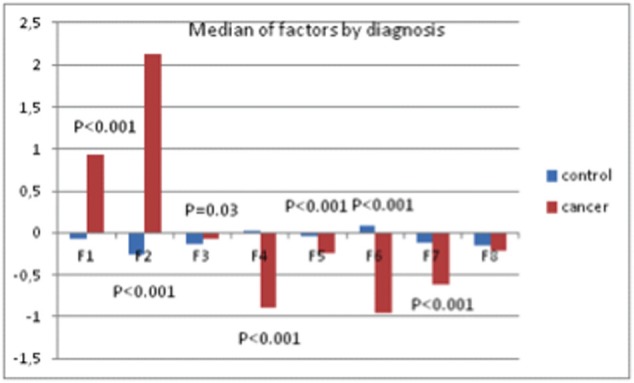
Median values of factors and *p*-values from multivariate ANOVA model.

The results showed a significant effect of the disease status on the individuated factors: multivariate analysis of variance (ANOVA) models including socio-demographic variables (age, parity, education, and marital status) and psychological treatment showed significant differences between patients and control healthy subjects (Figure 8), demonstrating that ALGA is a good instrument to be used to highlight those areas (factors) in which cancer patients were differentiated and to which the physicians should pay attention.

## 4. Intelligent optimisation for patient empowerment

### 4.1. Patient empowerment for doctors

Starting from the visualisation of ALGA results the physician will be able to concentrate on highlighted areas that are critical for patients’ management and that can affect their understanding and retention of medical information.

According to the example of the profile presented above, the physician will be presented with a brief verbal description of the patient’s psycho-cognitive condition similar to the following:

Patient X

Gender: female

Age: 45

Education Level: 18 years

Young age and level of education describe a patient with good abilities in paying attention and understanding complex information. However, a high score in cognitive closure is typical of a person who needs to find a fast solution to problematic situations. Tip: avoid goal- and problem-oriented information.

Modulating the way the information is provided will enhance patient empowerment through better understanding of the provided facts and consequently a more active role both in the decision-making process and in the behaviour the patients will adopt to cope with their illness. To ascertain for the effectiveness of patients’ profile on communication improvement patients’ empowerment will be measured in a future step of the project.

### 4.2. Patient empowerment for patients

In addition to empowering doctors, one of the key features of the IEmS is using interactivity to empower patients. Patient empowerment refers to the possibility of a patient to view data organised according to his/her perception of a domain, to retrieve patient-understandable information and, finally, to state a preferred decision. According to the National Research Council [7], health literacy involves the ‘degree to which individuals have the capacity to obtain, process, and understand basic health information and services needed to make appropriate health decisions’. Health literacy is not simply the ability to read. It requires a complex group of reading, listening, analytical, and decision-making skills, and the ability to apply these skills to health situations. For example, it includes the ability to understand instructions on prescription drug bottles, appointment slips, medical education brochures, doctor’s directions, and consent forms, and the ability to negotiate complex health-care systems.

It is known that a quality health-care outcome is associated with patients’ adherence to treatment. A literature review [8] on 569 studies reporting adherence to medical treatment from 1948 to 1998 showed that the average non-adherence rate is 24.8%. Martin *et al*. [9] measured patient’s adherence in clinical practice in a variety of ways including pill counts, self-reports or patient diaries, reports, and so on. In some disease conditions, more than 40% of patients sustain significant risks by misunderstanding, forgetting, or ignoring health-care advice. Realistic assessment of patients’ knowledge and understanding of the regimen, and their belief in it, will enable a more effective treatment and a better collaboration between physician and patient.

Adopting the lifestyle according to specific disease is commonly used in clinical practice. Leventhal *et al*. [10] describe an indicative example for colon cancer, which due to internal, cellular changes, may be seen to require procedures such as exercise to strengthen body resistance to encroaching tumours, a high-bran diet to remove carcinogenic poison from the gut, positive thinking to invigorate immune defences, and the seeking of specialised medical care. The authors proposed the common sense model of illness which provides an effective framework for the development of more detailed theories and models of behavioural processes involved in adaptation to episodes of physical and psychological disorders. The framework allows one to integrate factors at the level of the individual and the social system.

People facing serious illnesses such as cancer wish to take part in the decision-making process. Cancer is not only a danger for life but in addition treatments which are quite toxic can result in changes in body image and daily habits. Cancer screening may cause psychological distress, particularly when the results are not the expected. Patients are called to weigh the benefits and harms and inconveniences across options, so they are likely to experience personal uncertainty and to require support in decision making [11].

One of the most common causes of patient dissatisfaction is the feeling that they are not properly informed about (and involved in) their treatment.

Unfortunately, too often, clinicians lead patients to have a more passive and dependent role, hurting their self-confidence and undermining their ability to cope. When they are more active in the decision making, patients can better manage their feelings about their condition and will have better perspectives about their illness. When patients know that they can make a choice about their treatment, they may feel that they can overcome their illness.

The physician should guide the patient to set realistic goals about his/her treatment. This will give the patient a strong sense of self-efficacy that will encourage him/her to take on new, and maybe more difficult, tasks [12]. It is known that the majority of patients prefer to be involved in the decision making about their illness [2].

However, is not common for all individuals to want to play an active role in decision making. There are observed variables reported to affect patient preferences in communication with their physician, including gender, education level, age, life-threatening conditions, or cultural conditions [12, 13]. It should also be noted that patients are quite likely to vary in their preferences, depending on disease-specific conditions.

For instance, cancer patients with a more positive cancer prognosis may require a different amount of information than those who are facing a less hopeful prognosis [13].

To summarise, although some patients express low tolerance for high levels of information [13], more involved patients obtain better knowledge, have limited anxiety and feel more confident. What is more, informed patients have a better accurate perception of risk and seem to be less depressive [2].

Adopting software, used by patients, to the patient’s psycho-cognitive condition would improve his/her quality of life, the level of satisfaction with medical care, the emotional state but also the treatment procedure.

There are already numerous evaluation studies focusing on the functionality and the usability of PHR systems [14–26], and several evaluation models [26] incorporating intelligent factors, such as intelligent alerts, recommendations, and so on. In addition, there is a strong movement towards the following objectives [3]:

1. Improve the visual layout and style of the information from the medical record.
2. Create a use friendly design that makes it easier for the patient to manage their health.
3. Enable health professionals to more effectively understand and use patients’ health information.
4. Help family members and friends care for their loved ones.

There are quite a few techniques which could be used as a basis along with the patient profile constructed by the questionnaire and can be implemented within the Indivo X [11, 27], the PHR system that is used in the IEmS. Indivo X is a web based platform for patient centric integration of health-care information and for the development of patient driven applications to improve the quality, effectiveness, and convenience of the health-care system.

Apart from the integration and visualisation capabilities on health information sources across sites of care, Indivo X supports a flexible user-based policy which gives the power to the patient to indicate who has particular privileges on specific portion of his/her record. The patient has complete control over the sharing and distribution of his/her record [11]. The system allow patients to annotate any document in the record, update, hide, but not delete documents that are either out of date or that patients does not wish to share. Although Indivo X limits (disallows) content modification of records that were contributed by providers, the user is always free to add his/her personal opinion as annotations. With such privileges and restrictions, health-care providers are confident that verified data (e.g., lab results) were not altered by the patient and can also view annotations (patient’s opinion) for that data.

From the web-based interface, with a simple and easy to use mechanism, patients may share any type of data with another individual or class of individuals. Patient can (a) give to a specified person time-limited read-only access or (b) give access to all members of a group (e.g., a primary care practice). Indivo X also fully supports proxy roles. The adult child of an elderly parent may be the primary decision maker, and may therefore be granted full privileges by that parent. The same holds for parents of children and adolescents patients.

Transforming a PHR for easier use and more in tune with the user’s needs makes it more effective tool in personal health management. Below, we present possible techniques that we are currently exploring to be used in the IEmS.

#### 4.2.1. Adaptive Presentation

Adaptive presentation deals with how to present content in a manner that best suits individual users’ needs [28]. For example, the parts that are more interesting for the current user can be highlighted, or the description of the items returned can be adapted to play up the item’s features that are more relevant to the user’s needs, or changes to be more suitable to the user’s level of familiarity with the items. Understanding and recall are impaired if patients are anxious, if information is too detailed or too complex. For example, according to [2], it is suggested to give the most important information (e.g., advice that should be followed) at the beginning of the consultation and again at the end. If information is made specific, presented in categories and emphasised, it is more likely to be recalled and acted on.

The process of adapting content to specific user needs can be thought of as two main sub-processes. The first sub-process, which we will refer to as content adaptation, involves understanding what content can be most relevant to the current user’s interests, and how this content should be organised. The second sub-process, which we will refer to as content presentation, involves deciding how to most effectively present the selected content to the user.

Content adaptation of patient pages can be characterised along the following key dimension: the nature of the content provided as input. Along this dimension, there are two rather simple approaches in which adaptation is achieved by selecting appropriate canned pages or page fragments. These approaches are referred to in the literature as page and fragment variants respectively, and they have been extensively discussed in previous surveys (e.g., [28]). The latest years, however, more sophisticated approaches to content adaptation can be found, in which the input is abstract information.

Content presentation on the other hand present techniques that decide how to present content based on its relevance. Here, various techniques are employed to select the type of media most appropriate to deliver the content, given the interaction context and how to organise the selected fragments to be effectively communicated.

**Figure 9.**
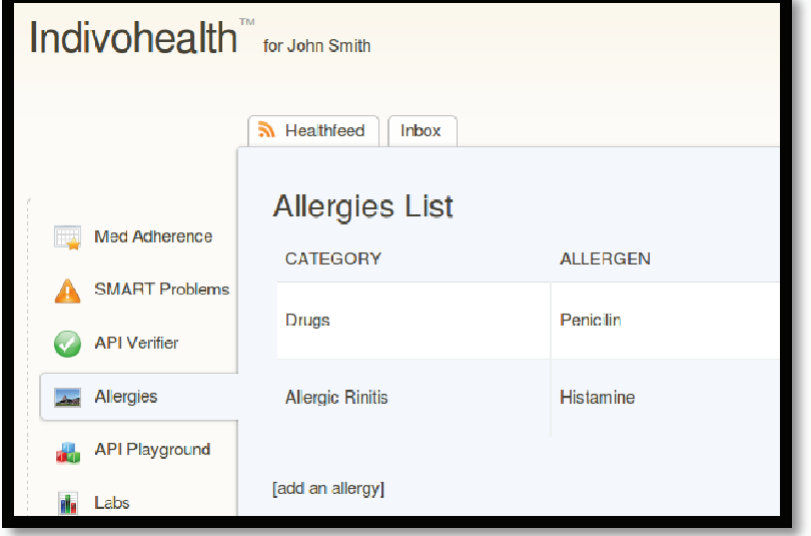
The list of allergies for a patient with high cognitive abilities.

**Figure 10.**
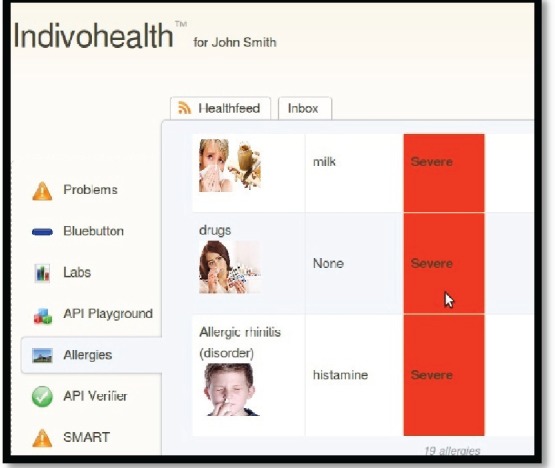
The list of allergies for a patient with low cognitive abilities.

As an example, consider the Figures 9 and 10 from IEmS, each one targeting a different user according to his level of cognitive aspects. On the first figure, more detailed information is presented for patient’s allergies whereas on the second one only a summary is presented accompanied with an image as well. Content representation also applies on menus according to the user profile/preferences.

#### 4.2.2. Adaptive Navigation

Adaptive navigation, on the other hand, comprises all of the ways to alter visible links to support hyperspace navigation [28]. The idea of adaptive navigation support techniques is to help users to find their paths in the web or in an application by adapting the way of presenting links to goals, knowledge, and other characteristics of an individual user.

Approaches in this area adaptively alter the appearance of links on every browsed page using methods, such as direct guidance, adaptive ordering, link hiding and removal, and adaptive link annotation. Adaptive navigation technologies, support personalised access to information. They have been evaluated in several application areas and have demonstrated their ability to let the users achieve their goals faster, reduce navigation overhead, and increase satisfaction [28].

**Figure 11.**
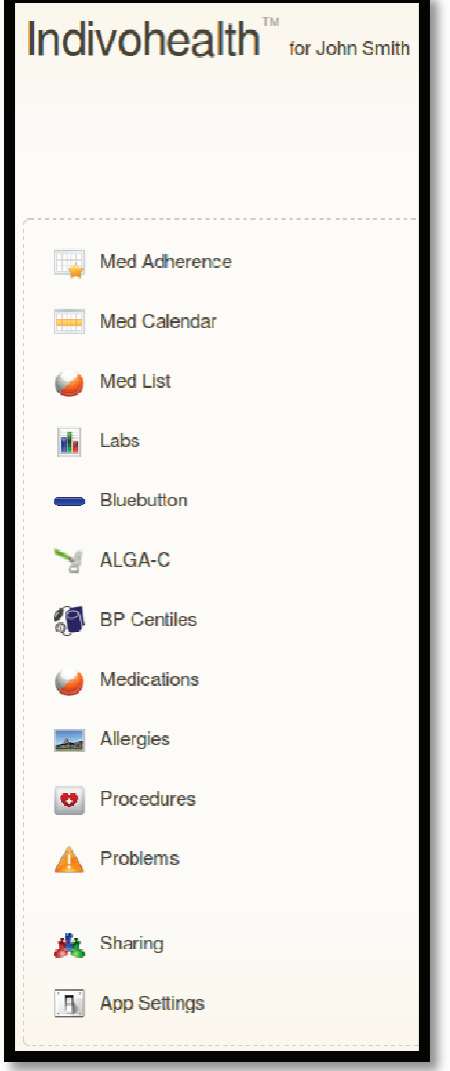
The navigation menu of a patient with high cognitive abilities.

**Figure 12.**
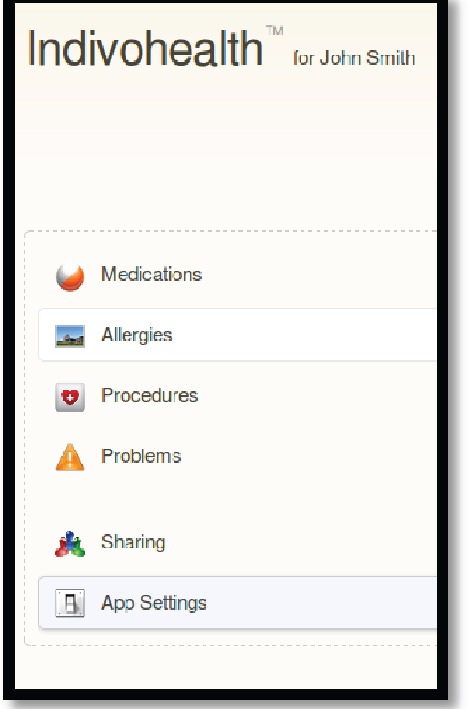
The navigation menu of a patient with low cognitive abilities.

An example scenario of adaptive navigation is shown in Figures 11 and 12. A patient with high cognitive abilities is able to choose among several options available, as shown on Figure 11. On the other hand, a patient with low cognitive abilities is not able to do easily. Therefore, only a list with the basic options is presented to him/her, as shown in Figure 12.

#### 4.2.3. Smart recommendations

Health information web sites offer a vast amount of information, but it can be difficult for the user to assess the reliability of the sources and the implications of the information. Several applications and web sites offer automated recommendations generated through a number of well-studied techniques. Those techniques include collaborative filtering, content-based techniques and knowledge-based approaches. In collaborative filtering, the system generates recommendations using only information about rating profiles for different users. Collaborative systems locate peer users with a rating history similar to the current user and generate recommendations using this neighbourhood.

Content-based techniques, on the other hand, generate a recommendation from the features associated with the presentation elements and the ratings that a user has given them. Finally, knowledge-based recommender approaches suggest topics based on inference about a user’s needs and preferences.

Web-based discussion and support groups can provide a more personal experience and add a social factor which can help patients with coping and commencing positive lifestyle changes. An indicative example is the Healthcare4Life [29], which combines the power of social networking with tele-health systems in empowering patients, especially seniors, to manage their health independently from home. Social networks can improve emotional health, which is essential for overall well-being. In addition, they can also help with motivating the patient, e.g., by achieving family support, or by doing monitoring task, and exercises together via a video link or in a virtual environment.

These techniques can deliver services that will recommend to the patient educational resources related to their condition and they will assist them in the in depth acknowledgment of their health status or disease to make informed decisions regarding their health care. Our PHR system will be able to process the patient data stored in the system and present useful links or explanatory content about the medical terminology which is related with the patient’s health status.

#### 4.2.4. Intelligent alerts

Intelligent alerts are yet another customisation technique planned to enhance the patient experience within IEmS. The system will be able to recognise an abnormality in the patient’s health status through the health information that the patient stores periodically in the PHR and suggest a doctor visit or alert the doctor in a critical situation. As the patient enters information about his status, medications, and so on, his health record will be instantaneously compared with a set of evidence-based rules to identify medical errors, quality issues, and hazards and will provide an instantaneous clinical alert. This real-time alerting capability is truly unique in the health-care industry where batch processing of data is the norm.

## 5. Conclusion

This paper presents the development and validation of the ALGA-C questionnaire and the ALGA-C profiler. The information delivered by ALGA has been analysed and statistically checked in an automated way. Results of ALGA enables physicians to rapidly inspect each patient’s individual cognitive profile and see at a glance the areas of concern.

In addition, an exploration on the techniques used for user interface optimisation according to patient profiles has been presented. Obviously, all of these different techniques complement each other and have the potential to improve the level of understanding of the medical information and, as a consequence, improve health, health care, and quality of life.

In addition to facing the challenges in customising a patient platform according to the individual needs, as future work, we intend to focus also on patient education. To this direction, the PHR system will provide an overview of the clinical procedures and will inform patients for the order of events that they should expect during their course of care. Another direction we could exploit is to facilitate more robust information exchange between individuals and their health-care providers.

Hopefully, IEmS platform will act as a decision support infrastructure, supporting the communication, interaction, and information delivery process between doctors and patients.

## Conflicts of interest

The authors have no conflicts of interest to declare.
